# Microarray platform consistency is revealed by biologically functional analysis of gene expression profiles

**DOI:** 10.1186/1471-2105-10-S11-S12

**Published:** 2009-10-08

**Authors:** Zhiguang Li, Zhenqiang Su, Zhining Wen, Leming Shi, Tao Chen

**Affiliations:** 1 Division of Genetic and Reproductive Toxicology, National Center for Toxicological Research, U.S. Food and Drug Administration, 3900 NCTR Rd., Jefferson, Arkansas 72079, USA; 2 Division of Systems Toxicology, National Center for Toxicological Research, U.S. Food and Drug Administration, 3900 NCTR Rd., Jefferson, Arkansas 72079, USA

## Abstract

**Background:**

Several different microarray platforms are available for measuring gene expression. There are disagreements within the microarray scientific community for intra- and inter-platform consistency of these platforms. Both high and low consistencies were demonstrated across different platforms in terms of genes with significantly differential expression. Array studies for gene expression are used to explore biological causes and effects. Therefore, consistency should eventually be evaluated in a biological setting to reveal the functional differences between the examined samples, not just a list of differentially expressed genes (DEG). In this study, we investigated whether different platforms had a high consistency from the biologically functional perspective.

**Results:**

DEG data without filtering the different probes in microarrays from different platforms generated from kidney samples of rats treated with the kidney carcinogen, aristolochic acid, in five test sites using microarrays from Affymetrix, Applied Biosystems, Agilent, and GE health platforms (two sites using Affymetrix for intra-platform comparison) were input into the Ingenuity Pathway Analysis (IPA) system for functional analysis. The functions of the DEG lists determined by IPA were compared across the four different platforms and two test sites for Affymetrix platform. Analysis results showed that there is a very high level of consistency between the two test sites using the same platform or among different platforms. The top functions determined by the different platforms were very similar and reflected carcinogenicity and toxicity of aristolochic acid in the rat kidney.

**Conclusion:**

Our results demonstrate that highly consistent biological information can be generated from different microarray platforms.

## Background

Microarray technology has become one of the most important approaches to globally reveal gene expression changes to identify genes associated with biological processes of interests. At present, several different microarray platforms are commercially available. The different platforms often have diverse types and various numbers of gene probes. Even for the probes corresponding to the same genes, there are usually various levels of binding affinity and specificity in different platforms. Therefore, questions are raised on whether the different microarray platforms can generate similar gene expression results [1-4]. To answer the questions, the MicroArray Quality Control (MAQC) project compared gene expression data generated from different microarray platforms for a set of common samples and found certain consistencies among the different platforms for the differentially expressed genes (DEG) [5,6].

Microarray methods are commonly used for examining thousands of genes simultaneously to reveal biological functional differences between examined samples. Therefore, biological functions should be considered as the ultimate endpoints for comparison of consistency across different microarray platforms. If different microarray platforms result in different DEGs but the same biological functions, we should acknowledge that these platforms are indeed comparable and reproducible because they tell us the same results. Although DEGs can be diverse when the experiments are performed at various times, in different laboratories or using dissimilar platforms, if most of the DEGs are true discoveries and enough of them are filled into distinct biological functional pathways, the altered biological functions can be identified. Therefore, it is logically hypothesized that high cross-platform concordance can be reached at biological function level.

In this study, we used the MAQC project data generated from kidney samples of rats treated with aristolochic acid (AA) [5]. AA is an active component of herbal drugs, but it is a nephrotoxin and carcinogen. A high prevalence of urothelial carcinoma was found in patients that developed nephropathy due to AA exposure [7-9]. Animal models also demonstrated that AA treatment resulted in renal failure in rodents [10], and tumors in the kidney, forestomach, and other tissues of rats and mice [11-13]. AA was identified among the most potent 2% of the carcinogens [14]. The International Agency for Research on Cancer (IARC) has classified products containing AA as human carcinogens [15]. Our previous studies have demonstrated that AA exposure produced DNA adducts and mutations in rat kidney [16,17] and induced carcinogenesis revealed by gene expression analysis of the same kidney samples [18]. If different microarray platforms are comparable for biologically functional analysis, we would expect these toxicological processes or functions to appear on the top functions altered in the AA treatment in each testing platform.

## Results

### Significant gene lists and common DEGs in different platforms

**Figure 1:**
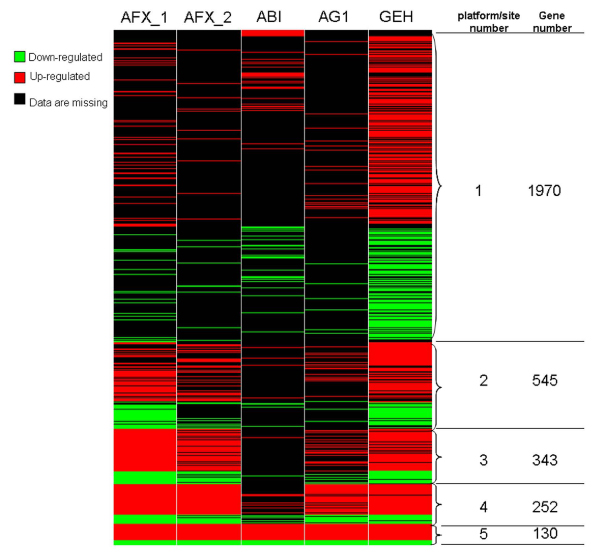
**Heat map of differentially expressed genes from 5 platforms/sites**. The green color indicates genes that are down-regulated; red color represents up-regulated genes; and black color means missing values. The platform number indicates in how many platforms a gene appears.

Unlike the MAQC study [5] in which the common genes across all platforms were used as a starting point for platform comparison, we used all genes in each platform microarray to independently select DEGs. Genes with *q *< 0.01 were considered as significantly changed. AA treatment of rats resulted in a number of DEGs in rat kidney, a carcinogenic target tissue of the carcinogen. The 1303 DEGs from Affymetrix test site 1 (AFX_1), 928 in Affymetrix test site 2 (AFX_2), 559 in Applied Biosystems site (ABI), 611 in Agilent (AG1), and 2346 in GE Health (GEH) were selected from 13713, 13713, 16280, 16135 and 12576 unique genes in each platform, respectively (See additional file 1: DEGs). Figure 1 is a heat map to compare the common DEGs from the different test sites. There are 130 DEGs common to all the five test sites, 252 DEGs common to four sites, 343 DEGs common to three sites, 545 DEGs common to two sites and 1970 present in only one site. The percentage of common DEGs in any two platforms is shown in Table 1. The percentages of common DEGs are low, ranging from 9% to 34%, except for the percentage between AFX_1 and AFX_2. The low percentages of common DEGs among the different platforms mainly resulted from the different gene probes in different platforms. There are only 5824 genes common to all the 4 platforms.

**Table 1:** Percentage of common differentially expressed genes between any two platforms/sites

	AFX_1	AFX_2	ABI	AG1	GEH
AFX_1	100				
AFX_2	52	100			
ABI	13	14	100		
AG1	29	34	16	100	
GEH	31	25	9	18	100

### Intra-platform comparison of biological processes

**Figure 2:**
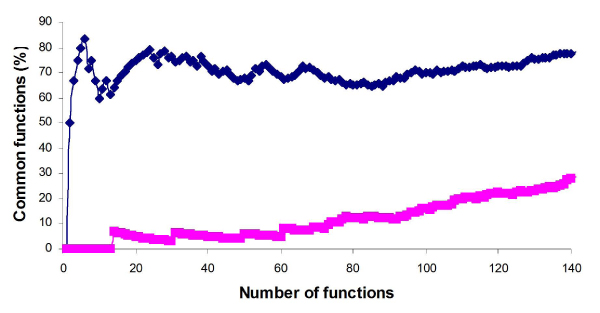
**Intra-platform overlaps of AA-altered biological functions**. Common AA-altered functions between the two function lists from AFX_1 and AFX_2 for a given number of functions are shown as blue color. The common functions between the control function lists generated from randomly selected genes for each of the test sites are displayed as red.

**Figure 3:**
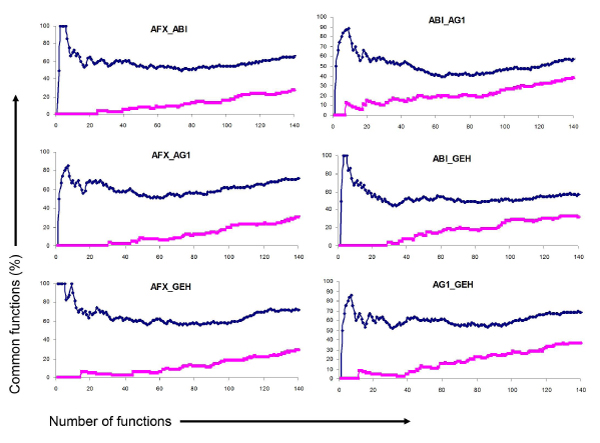
**Inter-platform overlap between any two platforms in terms of AA-altered biological functions**. The percentage of common functions is displayed against a given number of functions. The treatment is shown in blue and the randomly selected control is displayed in red. AFX, ABI, AG1 and GEH represent Affymetrix, Applied Biosystems, Agilent and GE Healthcare microarrays, respectively.

To compare the consistency of intra-platform microarray studies, two sets of data generated from two test sites for Affymetrix (AFX_1 and AFX_2) were used. The two DEG lists were imported to Ingenuity Pathway Analysis system (IPA) to identify the functions that were altered by AA treatment. The altered function processes are listed in Additional file 2. The two sets of function processes corresponding to the two DEG lists were compared and the percentages of common functions at a given number of functions are displayed in Figure 2. These two function lists exhibit high levels of overlap, whereas the control function lists from randomly chosen genes show much lower percentages of overlapping functions. The percentage of overlapping functions for the first 5 ranks are over 80%, and remains stable at about 70%. The control function lists, however, show very low concordance. There are no common functions between the control function lists for the top 10 functions, and the percentage of overlapping functions increases slightly when more functions are considered. Table 2 is a snapshot of the overlapping at the top 30 functions from Figures 2 and 3. The percentage of common functions between AFX_1 and AFX_2 is the highest (77%) among the comparisons, indicating that the intra-platform reproducibility for gene expression is better than inter-platform reproducibility in terms of biological functions.

**Table 2:** Percentage of common functions in the top 30 functions between any two platforms/sites

	AFX_1	AFX_2	ABI	AG1	GEH
AFX_1	100				
AFX_2	77 (3)	100			
ABI	57 (3)	53 (0)	100		
AG1	63 (0)	67 (3)	57 (10)	100	
GEH	70 (7)	63 (3)	47 (7)	63 (7)	100

Note: The numbers inside brackets represent the overlapping percentages of the top 30 functions from the control function lists.

### Inter-platform comparison of biological processes

Functional processes from the four platforms, ABI, AG1, GEH and AFX (AFX_1 was used for inter-platform comparison) were analyzed and compared. The percentages of overlapping AA-altered functions between any pair of two platforms vs. certain number of top functions are graphed and displayed in Figure 3. The patterns for the changes of common function ratio with number of top function are very similar in the six comparisons between the different platform microarrays. The highest concordance between platforms appear around top 10 functions, indicating that the major functional changes induced by AA treatment in rat kidney were commonly discovered by microarray analysis of gene expression from different platforms. The percentages of common functions at around top 50 of functions reach to the lowest and then slowly increase with function numbers in all of the comparisons. The percentages of common functions between the different platforms are from 47 to 70% while those between the control function lists are very low, from 0 to 10% (Table 2).

**Figure 4:**
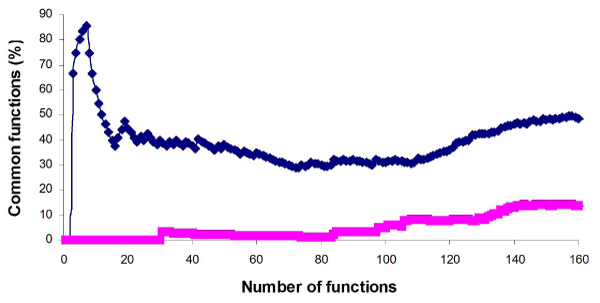
**Inter-platform overlap among all of the four platforms in terms of AA-altered biological functions**. The percentages of common functions are displayed against a given number of functions. The treatment is shown in blue and the randomly selected control is displayed in red.

A comparison across all of the 4 platforms also shows high concordance for the top 20 functions (45%, Figure 4), considering that only 4% DEGs (130 DGEs common to all four sites/3240 total DGEs that were used for the analysis) are common in the gene lists from the different platforms (Figure 1). In contrast, the percentage of common functions among the 4 platforms in the randomly selected controls is close to 0 when up to 80 of top functions are selected (Figure 4).

### Major biological processes changed by AA exposure in rat kidney

To examine whether each platform/site could reveal similar ongoing biological processes in rat kidneys exposed to AA, we compared the top 10 functions identified by IPA from the different platforms (Table 3). Since the major functions changed by a treatment normally appear to be the most significant, the top functions should reflect AA's carcinogenic characters in rat kidney. As expected, the major functions from all 5 platform/sites were carcinogenesis-related, such as cancer, tumor, neoplasm and tumorigenesis. Other functions like inflammatory disorder might reveal other toxicities of AA in rat kidney. When the top 20 functions were examined, carcinogenesis-related functions still remained as the main subject across all of the platforms (data not shown). In contrast, the top 10 functions derived from the control gene lists were diverse and inconsistent across the platforms/sites, and primarily were not cancer-related (Table 4).

**Table 3:** Cross-platform comparison of top 10 functions obtained from IPA analysis of differentially expressed genes identified from gene expression profiles in rat kidneys exposed to aristolochic acid

Rank	AFX_1	AFX_2	ABI	AG1	GEH
1	neoplasia	tumor	tumor	cancer	tumorigenesis
2	cancer	cancer	cancer	tumorigenesis	neoplasia
3	tumor	primary tumor	neoplasia	neoplasia	cancer
4	primary tumor	malignant tumor	primary tumor	tumor	tumor
5	malignant tumor	carcinoma	malignant tumor	primary tumor	primary tumor
6	carcinoma	inflammatory response	carcinoma	malignant tumor	malignant tumor
7	colorectal cancer	immune response	genetic disorder	carcinoma	carcinoma
8	inflammatory response	digestive organ tumor	digestive organ tumor	digestive organ tumor	inflammatory response
9	morphogenesis	dermatological disorder	inflammatory disorder	inflammatory disorder	colorectal cancer
10	immunological disorder	inflammatory disorder	colorectal cancer	immunological disorder	endometriosis

**Table 4:** Cross-platform comparison of top 10 functions obtained from IPA analysis of control gene lists that were randomly chosen from each platform

Rank	AFX_1	AFX_2	ABI	AG1	GEH
1	venous thrombosis	hereditary hemorrhagic telangiectasia	hypertension	nodule	psychological disorder
2	hypercalcemia	ulcerative colitis	movement	hematological disorder	schizophrenia
3	transmembrane potential	liver failure	Alzheimer's disease	adiposity	colitis
4	chemoattraction	movement	heart failure	insulin resistance	G-protein sigling, adenylate cyclase inhibiting pathway
5	defasciculation	arthritis	concentration	localization	neurotransmission
6	dicarboxylic aminoaciduria	dilation	hypoplasia	metabolic disorder	guidance
7	cancer	organization	lissencephaly	catabolism	cardiovascular process
8	tumorigenesis	progression	ataxia with oculomotor apraxia	unwinding	assembly
9	polyubiquitition	death	co-activation	permeability	decidualization
10	xeroderma pigmentosum	follicular carcinoma	diameter	abortion	fibrosis

## Discussion

The main purpose of gene expression studies using microarrays is to reveal the underlying biological differences between the two groups of samples. For example, examination of the lists of DEGs between controls and chemical-treated samples could reveal whether the chemical is toxic or not. DEG lists may change depending on the experiment, laboratory and platforms due to experimental variability, sample size, dissimilar probes in microarrays, different binding sites of probes for genes, and diverse methods for data normalization and selection. Therefore, DEG lists generated from identical RNA preparations could be considerably divergent if the microarray experiments are performed in different sites or with dissimilar microarray platforms [1,3]. The different DEG lists from various sites/platforms, however, still can be considered comparable if they reveal the same biological truth between the two groups of samples. It should also be pointed out that the higher percentage of overlapping functions compared to overlapping genes may be attributed to the fact that each function contains many genes.

In our analysis, the DEG lists identified from the platforms are very different because we did not use the common set of genes in the platforms for their selection. Only about 4% DEGs are common in all of the lists (Figure 1) and 9–34% DEGs are common between any two of the platforms (Table 1). In contrast, IPA analysis of these different lists resulted in high concordances of biological functions within one platform, between any two platforms and among all the platforms, especially for the top functions. The reproducibility of AA-altered functions for intra-platform is very high. This result is not unexpected because the microarrays tested in the different sites have the same gene probes and chemistries and the DEG lists from the two test sites are also similar. Although the percentages of common functions among the different platforms are a little bit lower than that for intra-platform, the concordances are still pretty high.

It is not unexpected to find the high concordance among the different platforms in terms of biological functions. Although false positive genes generally exist in a DEG list, most genes identified by microarrays are true DEGs when a high threshold method is used for the selection. When a biological process or function is changed, it normally results from the common effects of many genes. Therefore, even when dissimilar DEGs are present in platform analyses, they may be still involved in the same functions. Tan et al. compared the results from three commercial platforms and found that only 4 of the 185 unique genes were universal whereas the common top function, cell cycle, was identified by analysis of the top 200 genes from the different platforms [3]. This function reflects the differences in their samples, PANC-1 cells grown in serum-rich and serum-free media.

In this study, the rats were treated with AA using a similar protocol shown to result in the development of tumors in the rat kidney [12]. Six-week-old rats were treated with 10 mg AA/kg body weight five times a week for 12 weeks. The animals were sacrificed one day after the last treatment so that the gene expression could reflect the carcinogenicity and toxicity induced by AA in rat kidney. Although adenomas and adenocarcinomas in the renal cortex and papillomas in the renal pelvis were observed three months after the AA treatment in the carcinogenic study, atypical cells with gigantic nuclei and enlarged basophilic nucleoli in the tubular epiththelium of the renal cortex and multifocal dedifferentiation of the renal tubular epithelium and hyperplasia of the transitional epithelium in the renal pelvis were found immediately after the 3 month's treatment with 10 mg/kg AA. Therefore, the true biological differences between AA-treated and control samples are known. If a microarray analysis of these samples is reliable, it should reflect the pathogenesis exerted by AA treatment. The biological functions identified by the different sites or platforms reflect the biological effects of AA treatment (Table 3). The carcinogenesis-related functions appear in the top list in every laboratory site or platforms. This concordance suggests that data generated on different microarray platforms are comparable in terms of biological responses.

## Conclusion

In this study, we analyzed and compared microarray data generated by the MAQC project on AA-treated and control rat kidney samples from a biological function viewpoint. Our data demonstrated that there are high concordances of biological functions in intra- and inter-platform comparisons. Also, importantly, data from all the platforms or sites revealed the ongoing biological processes, carcinogenesis, in the kidneys of rats exposed to AA, a known carcinogen. These results suggest that highly consistent biological functions can be revealed when data from different microarray platforms are analyzed separately without restricting the analysis on a common set of genes found on all the platforms.

## Methods

### Microarray dataset

The microarray expression raw data were generated previously by the MAQC project to evaluate the consistency among different microarray platforms [5]. The total RNAs for microarray analysis were isolated from 12 kidney samples of Big Blue^® ^Transgenic Fisher 344 rats. Six of these rats were treated with 10 mg AA/kg body weight by gavage five times a week for 12 weeks while the other 6 rats were treated with the vehicle (0.9% sodium chloride). The treatment used induced DNA adducts, mutations and tumors in the kidneys of the AA-treated rats [12,16,17].

The microarray experiments were conducted at 5 test sites, including 2 sites using the AFX platform (Rat Genome 230 2.0). The other 3 sites used ABI (Rat Genome Survey Microarray), AG1 (Whole Rat Genome Oligo Microarray, G4131A) or GEH (Rat Whole Genome Bioarray, 300031) platforms. The total RNAs were isolated in our laboratory and distributed to each test site. Totally, 60 microarrays were used for AA-treated and control kidney samples at the 5 sites. Detailed information about animal treatment and microarray experiments can be found in an earlier publication [5].

### Selection of differentially expressed and randomly selected genes for functional analysis

The microarrays from the 4 platforms contain different numbers of probes for gene expression analysis. Specifically, 31099, 26857, 41071 and 35129 probes (or probe-set for AFX) are present in AFX, ABI, AG1 and GEH microarrays, respectively. According to Entrez gene ID, these probes correspond to 13713, 16280, 16135 and 12576 genes in AFX, ABI, AG1 and GEH microarrays, respectively. There are 5824 genes that are common across all 4 platforms. In contrast to the MAQC study that used the common genes for cross-platform comparison, we used the whole gene set of each platform to select DEGs. The data analysis was conducted using ArrayTrack software (http://www.fda.gov/nctr/science/centers/toxicoinformatics/ArrayTrack/index.htm). The gene expression data were normalized by using quantile method. Significance Analysis of Microarrays (SAM) was used to calculate *q* values. Two class unpaired analysis with 500 permutations was performed. DEGs were selected based on *q* < 0.01.

Mock gene lists were created by randomly selecting genes for each platform or site to serve as negative controls for cross-platform comparison. To generate the control gene lists, every probe of a platform was assigned an integer number as ID. The probes were randomly chosen by ID. The number of genes randomly chosen from a platform/site is the same as the number in the DEG list.

### Functional analysis

Functional analysis was performed using IPA online software. The genes in the DEG lists and the control gene lists were uploaded into IPA. The biological meanings for each gene list were interpreted using Core analysis, selecting rat for the species and the Relaxed (only molecules are filtered) for the filter. Both up- and down-regulated genes were analyzed. Databases corresponding to each individual platforms for AFX, ABI and AG1 and Ingenuity Knowledge Base (Gene only) for GEH were used for the analysis. A total of 1303, 928, 559, 611, and 2346 genes were uploaded and 1297, 924, 558, 610 and 2325 genes were eligible for function/pathway analysis for AFX_1, AFX_2, ABI, AG1 and GEH platforms/sites, respectively. In IPA, the functional relevance of a gene list was evaluated at 3 levels, category, function and function annotation. Each category includes multiple functions and each function usually includes multiple function annotations. A *p *value was calculated for each category/function/function annotation by IPA to show the enrichment of the input gene list in this category/function/function annotation. The *p *values are calculated using the right-tailed Fisher Exact Test by considering the number of analyzing genes that participating in that function and the total number of molecules that are known to be associated with that function in Ingenuity Knowledge Base [19]. Smaller *p *values indicate a higher level of enrichment in the input gene list of genes involved in the corresponding category/function/function annotation. The categories/functions/function annotations with *p *value < 0.05 were considered as significantly relevant with the analyzing genes and the top function annotations up to 500 were generated by the IPA. The functions related to these function annotations were selected for our analysis.

### The proportion of common functions identified by microarray analysis from the different platforms/sites

With IPA analysis, we generated 5 significant and 5 control category/function/function annotation lists. We focused on the function level for our analysis since similar results were found for analysis with function annotations. Because category is a sketchy level that has only a few of them for analysis, functions in this level have been eliminated from our analysis. Each function list was sorted according to *p *values. The repetitive functions in a function list were merged and their *p *values were averaged. The percentage of common functions between two or across all of the platforms/sites was calculated within a set of functions at a specific rank. For example, to calculate a percentage of common functions between platforms A and B at rank 50, the top 50 functions in both platforms were used for comparison and the number of the common functions divided by 50 would give the results. The same method was used to calculate the percentage of common functions across all of the platforms.

## Competing interests

The authors declare that they have no competing interests.

## Authors' contributions

ZL performed the data analysis and wrote the manuscript. LS participated in the critical discussion on the data analysis and manuscript preparation. ZS and ZW wrote computer programs needed for the data analysis. TC originally designed the study and was involved in the data analysis and writing the manuscript. All authors have read and approved the final manuscript.

## Supplementary Material

Additional file 1 Differentially Expressed Genes from 5 platforms/sites. Click here for file

Additional file 2 Categories/Functions/Function annotations generated in with Ingenuity Pathway Analysis. Click here for file
